# Gata3 Silencing Is Involved in Neuronal Differentiation and Its Abnormal Expression Impedes Neural Activity in Adult Retinal Neurocytes

**DOI:** 10.3390/ijms23052495

**Published:** 2022-02-24

**Authors:** Pei Chen, Yihui Wu, Jiejie Zhuang, Xuan Liu, Qian Luo, Qiyun Wang, Zihua Jiang, Anqi He, Shuilian Chen, Xi Chen, Jin Qiu, Yan Li, Ying Yang, Keming Yu, Jing Zhuang

**Affiliations:** State Key Laboratory of Ophthalmology, Zhongshan Ophthalmic Center, Sun Yat-sen University, Guangzhou 510000, China; chenpei@gzzoc.com.com (P.C.); wuyh45@mail2.sysu.edu.cn (Y.W.); zhuangjj7@mail2.sysu.edu.cn (J.Z.); kiffer@126.com (X.L.); luox97@mail2.sysu.edu.cn (Q.L.); wqiy@mail2.sysu.edu.cn (Q.W.); jiangzh36@mail2.sysu.edu.cn (Z.J.); angelho1227@163.com (A.H.); chenshlian@mail2.sysu.edu.cn (S.C.); chenx595@mail2.sysu.edu.cn (X.C.); qiuj27@mail.sysu.edu.cn (J.Q.); y13922236764@163.com (Y.L.); yangying856@163.com (Y.Y.)

**Keywords:** GATA binding protein 3, neuronal differentiation, retinal neurocytes, retina development, precursor cell

## Abstract

GATA binding protein 3 (Gata3), a zinc-finger transcription factor, plays an important role in neural development. However, its expression and bioactivity in the retina remain unclear. In the present study, our data indicated that Gata3 maintains the precursor state of 661W cells, and Gata3 silencing induces cell differentiation. The expression of Nestin, a marker of precursor cells, was significantly decreased in parallel, whereas the expression of Map2, a marker of differentiated neurons, was significantly increased following the decrease in Gata3. Neurite outgrowth was increased by 2.78-fold in Gata3-silenced cells. Moreover, Gata3 expression generally paralleled that of Nestin in developing mouse retinas. Both Gata3 and Nestin were expressed in the retina at postnatal day 1 and silenced in the adult mouse retina. Exogenous Gata3 significantly inhibited the neural activity of primary retinal neurocytes (postnatal day 1) by decreasing synaptophysin levels, neurite outgrowth, and cell viability. Furthermore, in vivo, exogenous Gata3 significantly induced apoptosis and the contraction of retinal outlay filaments and decreased the a- and b-waves in adult mouse intravitreal injected with AAV-Re-Gata3-T2A-GFP. Thus, Gata3 silencing promotes neuronal differentiation and neurite outgrowth. Its abnormal expression impedes neural activity in adult retinal neurocytes. This study provides new insights into Gata3 bioactivity in retinal neurocytes.

## 1. Introduction

GATA binding protein 3 (Gata3) is a zinc-finger transcription factor involved in the embryonic development of various cell types (e.g., fat cells, neural crest cells, and lymphocytes) and tissues (kidney, liver, brain, spinal cord, and mammary gland) [1,2,3]. During central nervous system (CNS) development (the first 2 weeks of postnatal development), an early study indicated that the Gata3 mRNA was expressed in the intergeniculate leaflet, ventral lateral geniculate nucleus, pretectal nucleus, nucleus of the posterior commissure, superior colliculus, inferior colliculus, periaqueductal gray, substantia nigra and raphe nuclei [4]. As postnatal development progresses, however, Gata3 expression decreases, and a faint signal is observed in these regions of the adult CNS [5,6]. Zhao and colleagues indicated that Gata3 is mainly expressed in developing NeuN-positive, not GFAP-positive, cells [4]. It is also associated with early motor neuron and interneuron precursors [7].

The retina is one part of the CNS with a delicate structure [8]. However, discrepancies in the Gata3 expression profile during retinal development have been reported. For example, Maeda et al. indicated that Gata3 only regulates lens development and is not expressed in the retina of embryonic and postnatal eyes [9]. However, Martynova et al. found that GFP was expressed from a 5 kb Gata3 promoter in the developing lens with additional activity in the retina, suggesting that Gata3 might be expressed in the early stage of retinal development [10]. Moreover, Celikkaya et al. reported that Gata3 promoted the neural progenitor state [11,12]. Neural progenitor cells are present in the postnatal retina [13,14]. Nestin, a specific marker of neural progenitor cells, is expressed in the retina at E13. Subsequently, its expression expands distally to P3 [15]. Therefore, this evidence further suggests that Gata3 might be expressed in the postnatal retina, which requires further investigation.

As early as 1995, Pandolfi et al. reported that homozygous Gata3 mutant mice are invariably lethal at approximately days 11–12 of embryogenesis. Massive internal bleeding, marked growth retardation, and gross aberrations in fetal liver hematopoiesis are observed [16]. Moreover, Gata3 loss induces severe deformities of the brain with complete ventricular collapse and spinal cord damage with extensive neural tube destruction [16,17]. Subsequent studies revealed that blocking Gata3 activity inhibits the proliferative and neurogenic ability of zebrafish neural stem cells in the telencephalon [12]. However, the effect of Gata3 on the mature CNS remains controversial. For example, injury results in Gata3 re-expression in glia and newborn neurons in 6-month-old zebrafish [12,18]. The authors suggested that Gata3 plays a key role in reactive proliferation of the progenitors, reactive neurogenesis, and migration of newborn neurons [19,20,21]. Gata3 was identified as an essential factor for regenerative capacity in vertebrates [6]. Moreover, in neurodegenerative diseases, Zhou suggested that Gata3 functions as a neuroprotective factor in 6-OHDA-treated neural cells and might become a novel molecular therapy target in Parkinson’s disease [22]. However, a recent study reported different findings. Gata3 promoted the neural progenitor state but not neurogenesis [11]. Therefore, the bioactivity of Gata3 in mature retinas also requires further investigation.

Using a retinal precursor cell line and primary postnatal retinal cells, we investigated the Gata3 expression profile and its bioactivity in proliferation, differentiation, and neurite outgrowth through gene interference to address these questions. Moreover, the in vitro results were also confirmed in vivo. Scotopic electroretinogram (ERG) was employed to examine the function of retinal neurons in the adult mouse.

## 2. Results

### 2.1. Gata3 Silencing Induces Differentiation and Reduces the Viability of 661W Cells

**Figure 1:**
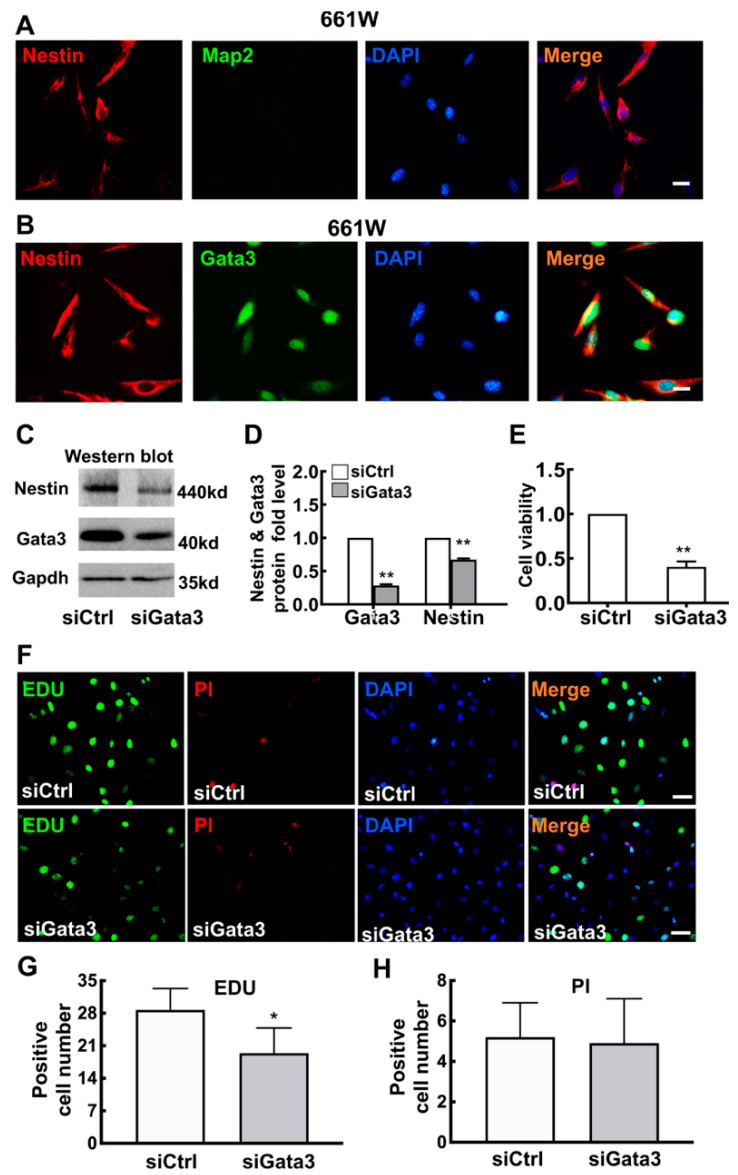
Gata3 silencing induces differentiation and reduces the viability of 661W cells. (**A**) 661W cells were positive for Nestin (red, in the cell cytoplasm) and negative for Map2. The nucleus is labeled with DAPI (blue). (**B**) Gata3 was expressed at high levels in 661W cells (green, in cellular nuclei). (**C**), Gata3 was silenced in 661W cells by an siRNA. Nestin expression decreased in parallel, as evidenced by the Western blot results. (**D**) The relative levels of the Gata3 and Nestin proteins in 661W cells are presented in a histogram. (**E**) Gata3 silencing significantly reduced the viability of 661W cells. Scale bars represent 10 μm. (**F**) The EDU signaling was much weaker in Gata3 silenced cells (green, nuclear). No obvious difference in PI staining was observed in two groups (red, nuclear). The nucleus is labeled with DAPI (blue). Scale bars represent 20 μm. (**G**) The EDU-positive cell number in 661W cells is presented in a histogram. (**H**) The PI-positive cell number in 661W cells are presented in a histogram. The asterisks indicate statistically significant differences (* p < 0.05, ** *p* < 0.01).

According to previous studies, 661W cells are a cone-photoreceptor-specific precursor-like cell line [23,24]. Here, 661W cells were used as an ideal tool to investigate the differentiation of retinal neurocytes. As shown in Figure 1A, 661W cells were characterized with stem cell markers. Staining for Nestin (red, in cell cytoplasm), a marker of precursor cells, was positive, and staining for Map2, a marker of differentiated neurons [25], was negative in 661W cells. Moreover, Gata3 was expressed at high levels in the nuclei of 661W cells (green, Figure 1B).

Gata3 was silenced in 661W cells using an siRNA to investigate its bioactivity in retinal cell differentiation. As shown in Figure 1C, Nestin expression decreased in parallel with Gata3 silencing in 661W cells. The relative levels of the Gata3 and Nestin proteins in 661W cells are presented in a histogram showing that Gata3 was downregulated by 72% in Gata3 siRNA-treated 661W cells (siControl, 1; siGata3, 0.28 ± 0.02, ** *p* < 0.01, Figure 1D). Gapdh was used as an internal control. Nestin expression was also decreased by 38% (siControl: 1; siGata3: 0.67 ± 0.02, ** *p* < 0.01, Figure 1D). The CCK-8 assay also indicated that Gata3 silencing significantly reduced cell viability (siControl, 1; siGata3, 0.406 ± 0.06, ** *p* < 0.01, Figure 1E). Moreover, we further performed the 5-ethynyl-2′-deoxyuridine (EDU) and Propidium iodide (PI) Nucleic Acid Staining to label the proliferating cell and dead cell, respectively. Our data showed that Gata3 silencing by siRNA impedes cell proliferation (EDU, green, cell nucleus, siCtrl: 28.7 ± 4.62, siGata3: 19.4 ± 5.44, * *p* < 0.05, Figure 1F,H), with no obvious impact on cell death (PI, red, nucleus, siCtrl: 5.2 ± 1.7, siGata3: 4.9 ± 2.2, *p* > 0.05, Figure 1F,H), which explains the decreasing cell viability in 661W cells.

**Figure 2:**
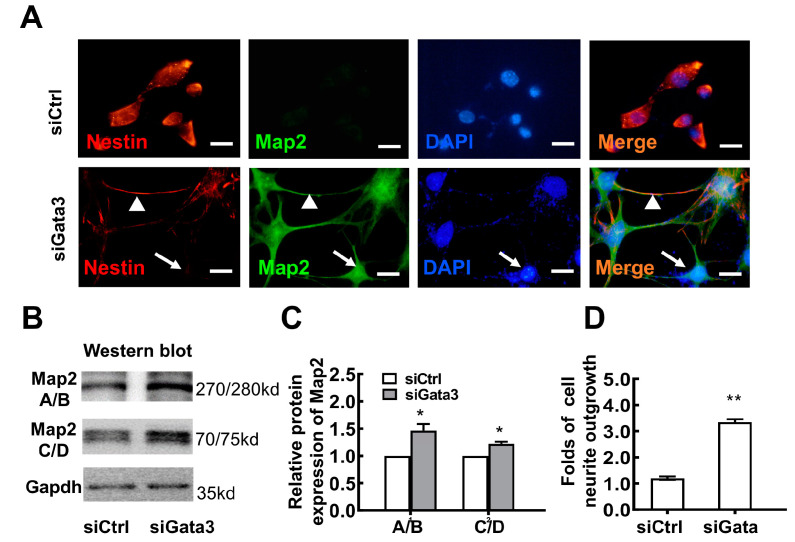
Gata3 silencing induces the differentiation of 661W cells. (**A**) Gata3 silencing by an siRNA decreased Nestin expression (red, in the cell cytoplasm) while upregulating Map2 expression in 661W cells (green, in the cell cytoplasm). (**B**) Gata3 silencing profoundly increased the expression of both Map2 (A/B) and Map2 (C/D). (**C)**, The relative levels of the Map2 (A/B) and Map2 (C/D) proteins in 661W cells are presented in histograms. (**D**) Gata3 silencing significantly promoted retinal neurite outgrowth from 661W cells. Scale bars represent 10 μm. The asterisks indicate statistically significant differences (* *p* < 0.05, ** *p* < 0.01).

Double staining for Nestin (a marker of precursor cells) and Map2 (a marker of differentiated neurons) was performed to further characterize the differentiation status of Gata3-silenced cells. As shown in Figure 2A and Supplemental Data S1A, the scrambled control siRNA did not affect the expression of Nestin in 661W cells, which showed red staining in the cytoplasm with no obvious Map2 staining. However, Gata3 silencing induced Map2 expression and downregulated Nestin expression in 661W cells (Figure 2A and Supplemental Data S1B), implying that Gata3 silencing promotes cell differentiation. More importantly, Nestin was almost completely silenced in the fully differentiated cells that presented fully developed neurites and strong Map2 staining (green, white arrows, Figure 2A). These Map2-positive cells obviously displayed a neuronal morphology with smaller somas and longer axons. In addition, poorly differentiated cells with both Map2 and Nestin positivity were observed. Map2 staining was detected in the cytoplasm, whereas Nestin staining was detected in neural axons of the same cell (red, white arrowhead, Figure 2A). This phenomenon might be due to the varying Gata3 siRNA knockdown efficiency in single cells. As shown in Supplemental Data S1B, significant decreased Nestin (green) and increased Map2 staining (green) were observed in the Gata3-silenced cells, with distinguishable neurites (white arrows). While, in the cells that still expressed Gata3 (red, cell nuclear), poorly differentiated cells with both Map2 and Nestin positivity were observed (white arrowheads). Together, this evidence strongly suggests that the Gata3 expression level and cell differentiation levels are inversely proportional.

In addition, the Western blot data further indicated that the 661W cells treated with scrambled control siRNA only showed weak expression of Map2 (A/B) and Map2 (C/D) (Figure 2B). Gata3 silencing profoundly increased the expression of Map2 (A/B) and Map2 (C/D). The relative levels of the Map2 (A/B) and Map2 (C/D) proteins were quantified using densitometry, normalized to Gapdh levels, and then presented in a histogram (Figure 2C). The expression of both Map2 (A/B) and Map2 (C/D) was significantly increased in Gata3-silenced 661W cells compared with control cells (A/B: siControl, 1; siGata3, 1.46 ± 0.12; C/D: siControl, 1; siGata3, 1.22 ± 0.04; * *p* < 0.05). Moreover, Gata3 silencing significantly promoted retinal neurite outgrowth in 661W cells (siControl, 1.204 ± 0.067, siGata3, 3.346 ± 0.114, ** *p* < 0.01, Figure 2D). Collectively, these observations indicate that Gata3 silencing may be involved in the mechanism regulating neuronal differentiation and neurite outgrowth.

**Figure 3:**
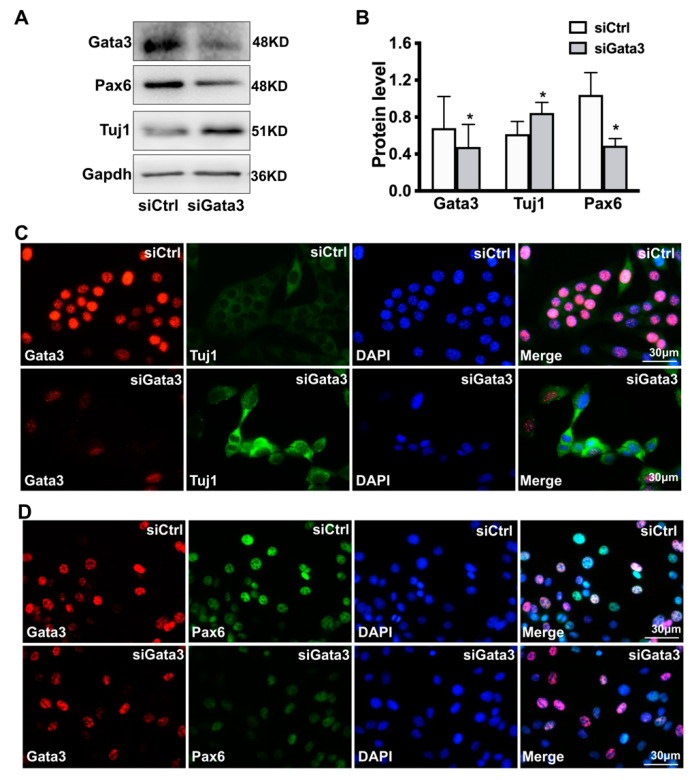
Gata3 silencing promotes differentiation in 661W cells. (**A**) Gata 3 silencing decreases the expression of pax6 while increases the expression of Tuj1 in 661W cells, as evidenced by Western blot. (**B**) The relative levels of the Gata3, pax6, and Tuj1 proteins in 661W cells are presented in a histogram. (**C**) Significant up-regulation of Tuj1 (cytoplasm, green) were observed in the Gata3-silenced cells (cell nucleus, red). (**D**) Decreased pax6 staining (cell nucleus, green) was observed in the Gata3-silenced cells (cell nucleus, red). The nucleus is labeled with DAPI (blue). Scale bars represent 30 μm (* *p* < 0.05).

Not only that, as shown in Figure 3A,B, Gata 3 silencing decreased the expression of pax6 (a neuronal progenitor marker) while increased the expression of class III beta-tubulin (Tuj1, a neuron marker) in 661W cells, as evidenced by Western blot (for Gata3, siCtrl: 0.68 ± 0.34, siGata3: 0.48 ± 0.24; for pax6, siCtrl: 1.04 ± 0.24, siGata3: 0.49 ± 0.08; for Tui1, siCtrl: 0.62 ± 0.14, siGata3: 0.84 ± 0.12, * *p* < 0.05). Moreover, results obtained from double immunostaining assay also showed stronger signaling of Tuj1 (cytoplasm, green) and fainter pax6 signaling (cell nucleus, green) in the Gata3-silenced cells (cell nucleus, red), compared with that of control cells, further suggesting its role in neuron differentiation.

### 2.2. The Gata3 Expression Profile in the Developing Mouse Retina Is Generally Similar to That in Precursor-Like Cells

**Figure 4:**
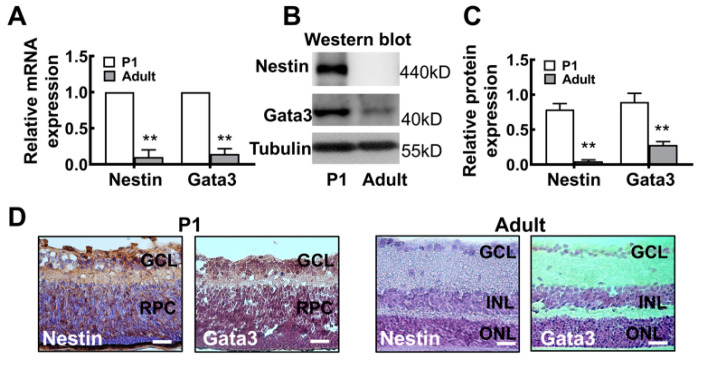
The Gata3 expression profile in the mouse retina. (**A**) The mRNA levels of Nestin and Gata3 were significantly higher in P1 mouse retinas than in adult mouse retinas, as determined using real-time PCR. (**B**) Both Nestin and Gata3 were expressed at high levels in P1 mouse retinas and absent in adult mouse retinas, as evidenced by Western blotting. (**C**) The relative levels of the Nestin and Gata3 proteins in the mouse retina are presented in histograms. (**D**) Both Nestin and Gata3 were expressed at high levels in all layers of the P1 mouse retinas but weakly expressed in adult mouse retinas. Scale bars represent 50 μm. The asterisks indicate statistically significant differences (** *p* < 0.01).

We first quantitatively examined the patterns of Nestin and Gata3 mRNA and protein expression in the mouse retina to further verify the in vitro results. As shown in Figure 4A, levels of the Nestin and Gata3 mRNAs were significantly decreased in the adult retina (Nestin: P1, 1; adult, 0.102 ± 0.098. Gata3: P1, 1; adult, 0.144 ± 0.072. ** *p* < 0.01). Similarly, the protein levels were consistent with mRNA levels (Figure 4B). The relative intensities of the bands obtained from Western blots were quantified using densitometry and normalized to tubulin levels. Nestin was expressed at high levels in postnatal day 1 (P1) mouse retinas and absent in adult mouse retinas (P1, 0.79 ± 0.08; adult, 0.05 ± 0.02; ** *p* <0.01), indicating the lack of differentiation of retinal cells in P1 mouse. Gata3 expression was noticeably increased after birth, as intense bands were detected in the lysates of P1 mouse retinas; however, very weak expression was observed in adult mouse retinas (P1, 0.90 ± 0.12; adult, 0.28 ± 0.05. ** *p* < 0.01; Figure 4B,C).

Moreover, the localization of Nestin and Gata3 in the mouse retina was analyzed using immunohistochemistry. The strong staining for Nestin in P1 mouse retinas and faint staining in adult mouse retinas indicated that P1 mouse retinal neurons were still immature; however, these cells were fully differentiated in adult mouse retinas (Figure 4D). Moreover, Gata3 was predominantly distributed in the ganglion cell layer (GCL) and inner nuclear layer (INL) of the immature P1 neonatal mouse retina, with some protein detected in the innermost part of the retinal progenitor cell layer (RPC). No distinct immunoreactive cells were observed in differentiated retinal neurons of adult mice. Taken together, this evidence reveals similar expression patterns of Nestin and Gata3 in the mouse retina, further suggesting a regulatory relationship between the differentiation and maturation of retinal neurons.

### 2.3. Exogenous Gata3 Inhibits Synaptophysin (Syn) Expression and Neurite Outgrowth and Reduces the Viability of Primary Retinal Neurocytes in Vitro

**Figure 5:**
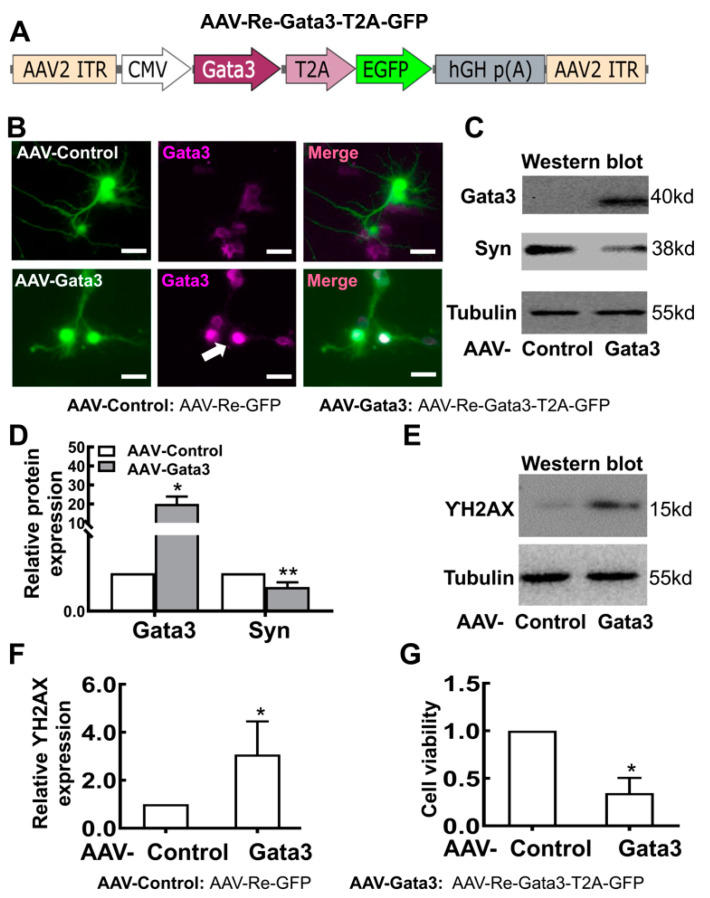
Exogenous Gata3 partially inhibits the maturation of primary retinal neurocytes in vitro. (**A**) The AAV-Re-Gata3-T2A-GFP adenovirus construct. (**B**) At 5 days after infection, GFP (green) was expressed at high levels in primary retinal neurocytes infected with AAV-Re-Gata3-T2A-GFP or AAV-Re-GFP. Exogenous Gata3 (amaranth, white arrows) was expressed in the nuclei of primary cells infected with AAV-Re-Gata3-T2A-GFP. (**C**) Exogenous Gata3 significantly increased Syn expression in primary retinal neurocytes. (**D**) The relative levels of the Gata3 and Syn proteins in primary retinal neurocytes are presented in histograms. (**E**) Gata3 overexpression induced a significant increase in γH2AX levels. (**F**) The relative level of the γH2AX protein in primary retinal neurocytes is presented in a histogram. (**G**) Gata3 overexpression significantly reduced the viability of primary retinal neurocytes. Scale bars represent 10 μm. The asterisks indicate statistically significant differences (* *p* < 0.05, ** *p* < 0.01).

As we described above, the Gata3 pathway is involved in the differentiation of retinal cell lines. What occurs in primary retinal neurocytes overexpressing Gata3? Primary retinal neurocytes (P1) were infected with the adenovirus AAV-Re-Gata3-T2A-GFP (Figure 5A) or AAV2-Re-GFP [26]. At 5 days after infection, GFP was expressed at high levels in cells (green). The cells were fixed and stained with a Gata3 antibody. As shown in Figure 5B, exogenous Gata3 (amaranth, white arrows) was located in the nuclei of primary cells. Western blot assays indicated a more than 20-fold increase in Gata3 levels in primary retinal neurocytes infected with AAV-Re-Gata3-T2A-GFP compared with cells infected with AAV-Re-GFP (AAV-Re-GFP, 1; AAV-Re-Gata3-T2A-GFP, 20.06 ± 3.84; * *p* < 0.05, Figure 5C,D). Moreover, the level of synaptophysin (Syn), which is involved in synapse formation [27,28], was significantly decreased in the cells infected with AAV-Re-Gata3-T2A-GFP compared with that in cells infected with AAV-Re-GFP (AAV-Re-GFP, 1; AAV-Re-Gata3-T2A-GFP, 0.64 ± 0.12; ** *p* < 0.01, Figure 5C,D). Consistent with the results from the cell line, this evidence indicated that re-expression of Gata3 might inhibit the viability of primary postnatal retinal neurocytes.

Furthermore, DNA instability is a characteristic of neurocytes [29]. Therefore, we analyzed the levels of γH2AX, a marker of DNA double-strand breaks [30,31], using Western blotting. As shown in Figure 5E,F, Gata3 overexpression induced a significant increase in γH2AX levels (AAV-Re-GFP, 1; AAV-Re-Gata3-T2A-GFP, 3.07 ± 1.37; * *p* < 0.05), which indicates an increase in DNA double-strand breaks in neurocytes expressing exogenous Gata3. Indeed, exogenous Gata3 noticeably reduced cell viability, as evidenced by CCK-8 assay (AAV-Re-GFP, 1; AAV-Re-Gata3-T2A-GFP, 0.345 ± 0.16; * *p* < 0.05) (Figure 5G), which is not consistent with the results obtained from the 661W cell line.

### 2.4. Gata3 Overexpression Impairs Mouse Retinal Function in Vivo

**Figure 6:**
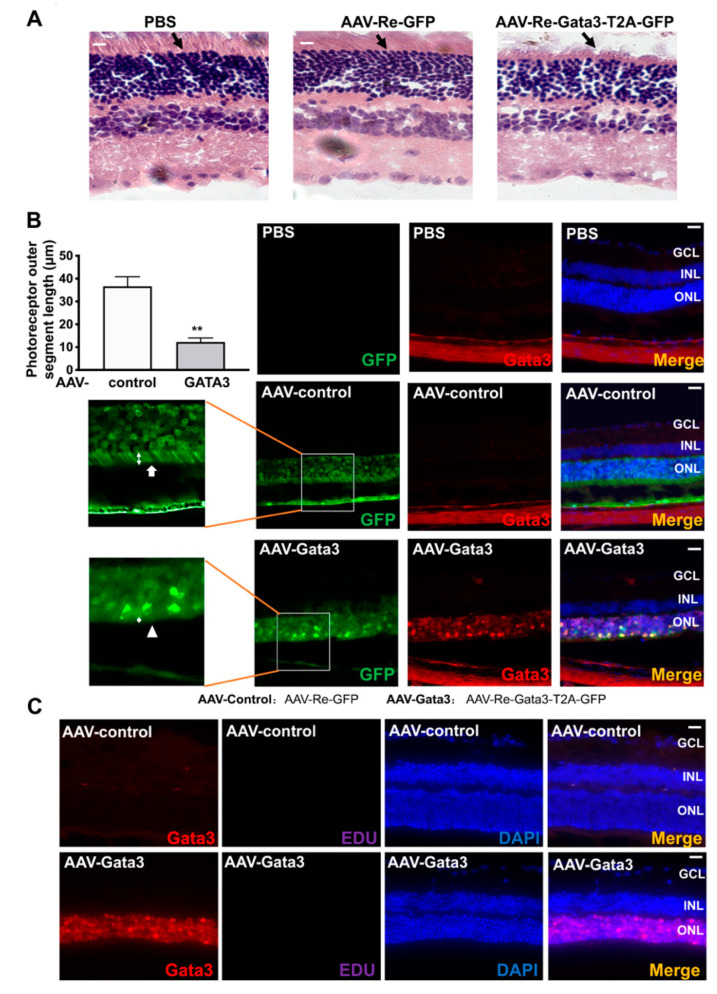
Gata3 overexpression induced serious damage in the mouse retina in vivo. (**A**) Exogenous Gata3 induced the contraction of the outer segment of photoreceptors in the mouse retina, as evidenced by HE staining (*n* = 6 for each group). (**B**) Mice infected with AAV-Re-Gata3-T2A-GFP (green) exhibited strong staining for Gata3 (cell nucleus, red) in the ONL of the retina, which was not observed in retinas infected with AAV-Re-GFP or PBS (*n* = 6 for each group). The outer segment of photoreceptors (white arrows) was clearly observed in the retina expressing GFP alone, whereas it was contracted in the retina expressing exogenous Gata3 (white arrowheads). (**C**) No obvious EDU signaling was detected in the adult mouse retina neither infected with AAV-Re-GFP nor AAV-Re-Gata3-T2A-GFP (*n* = 6).** *p* < 0.01. Scale bars represent 50 μm.

AAV-Re-Gata3-T2A-GFP was used to infect the right eyes of adult mice (*n* = 8) through intravitreal injection to further explore the physiological role of Gata3 in the retina in vivo. The vitreous of the left eyes of each mouse (*n* = 8) were injected with a control adenovirus (AAV-Re-GFP) as self-control. Six eyes from 6 mice were intravitreally injected with PBS (2 μL) as a negative control. At 1 and 3 months after adenovirus infection, the mouse eyes were fixed and analyzed using HE staining. Our data showed that most GFP-positive cells in the mouse retinal were concentrated in the outer nuclear layer retina (ONL), and positive for Recoverin, a photoreceptor marker (Supplemental Data S2). Very notably, exogenous Gata3 induced serious damage in the outer segment of photoreceptors (black arrows, Figure 6A) at 3 months after infection, which was not observed in the retina at 1 month (data not shown). Moreover, immunofluorescence staining also confirmed this finding. As shown in Figure 6B, green fluorescence was observed in the ONLs of mouse retinas infected with AAV-Re-Gata3-T2A-GFP or AAV-Re-GFP, but absent in the mouse retina injected with PBS (*n* = 6 for each group), indicating that the adenovirus specifically infected cells in the ONLs of the retina. Retinal cells infected with AAV-Re-Gata3-T2A-GFP (green) exhibited strong staining for Gata3 (cell nucleus, red) in the ONL of the mouse retina. The photoreceptor outer segmental length was measured with ImageJ (LOCI, University of Wisconsin, Madison, WI, USA), and ten serial slides of each eye (6 eyes in each group) were taken into statistical analysis, and in each slide the thickness of the three thickest positions was measured. Similarly, the outer segment of photoreceptors (white arrows) was clearly observed along ONLs in the retina expressing only GFP (34.64 ± 4.16 μm), whereas it was contracted in the retina expressing exogenous Gata3 (12.29 ± 1.74 μm, * *p* < 0.05, white arrowheads, Figure 6B). However, exogenous Gata3 did not induce neurogenesis, as detected using EDU staining (Figure 6C), which is not consistent with previous studies [12].

**Figure 7:**
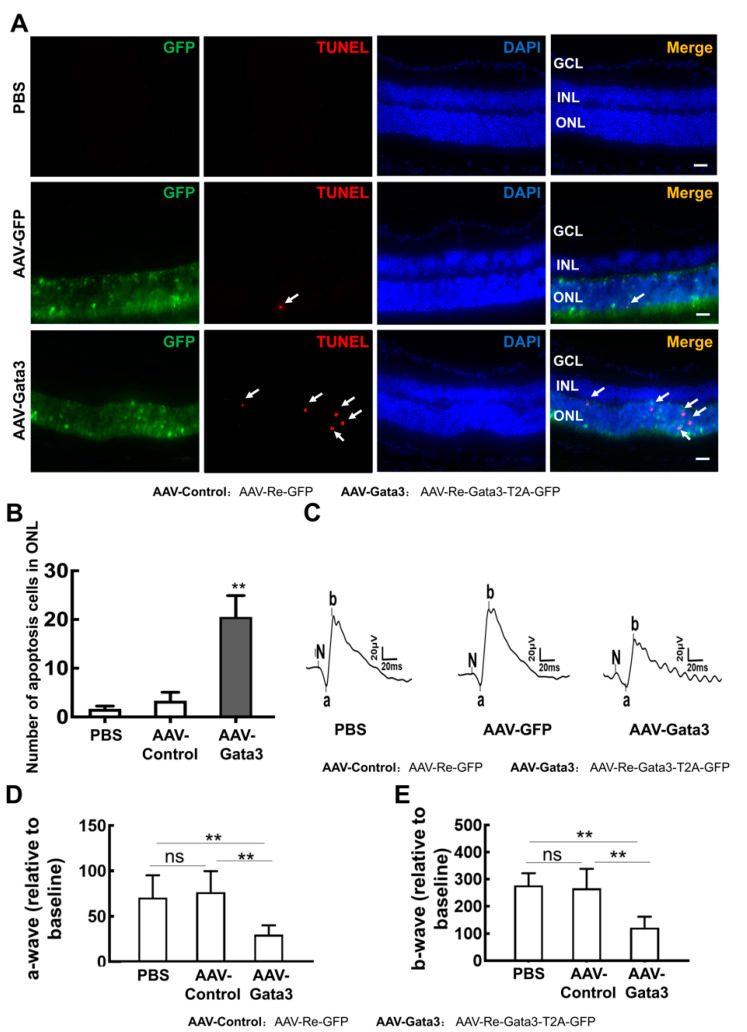
Gata3 overexpression induced serious damage in the mouse retina in vivo. (**A**) Exogenous Gata3 remarkably induced apoptosis (white arrows) in the ONL of the retina. (**B**) Number of apoptotic cells in ONL of retinas infected with AAV-Re-Gata3-T2A-GFP, control vector, and PBS (*n* = 6 for each group). (**C**) AAV-Re-GFP infection did not affect retinal function. However, the amplitudes of both the a- and b-waves were decreased in eyes infected with AAV-Re-Gata3-T2A-GFP (*n* = 6 for each group). (**D**) The amplitudes of the a-wave in mice with different treatments are presented in histograms (*n* = 6 for each group). (**E**) The amplitudes of b-waves in mice with different treatments are presented in histograms (*n* = 6 for each group). The asterisks indicate statistically significant differences (** *p* < 0.01). ns indicates that the differences were not statistically significant. Scale bars represent 50 μm.

Very importantly, our data showed that exogenous Gata3 remarkably induced apoptosis in the ONL (Figure 7A). Significantly more apoptotic cells (white arrows) were observed in the ONL of retinas expressing Gata3 than in the control retinas (PBS,1.2 ± 1.1; AAV-Re-GFP, 3.33 ± 1.73; AAV-Re-Gata3-T2A-GFP, 20.53 ± 4.38; *n* = 6, ** *p* < 0.01) (Figure 7B). Moreover, the retinal damage induced by exogenous Gata3 was confirmed by the ERG results. As shown in Figure 7C, AAV-Re-GFP infection did not alter retinal function compared with mice intravitreally injected with PBS. However, the amplitudes of both the a- and b-waves were significantly decreased in eyes infected with AAV-Re-Gata3-T2A-GFP compared with eyes injected with AAV-Re-GFP (for the a-wave, PBS: 70.43 ± 24.74; AAV-Re-GFP: 76.50 ± 17.60; AAV-Re-Gata3-T2A-GFP: 29.75 ± 10.43. *n* = 6, ** *p* < 0.01, Figure 7D. For the b-wave, PBS: 276.8 ± 45.04; AAV-Re-GFP: 267.0 ± 27.00; AAV-Re-Gata3-T2A-GFP: 121.9 ± 40.12. *n* = 6, ** *p* < 0.01, Figure 7E). Taken together, re-expression of Gata3 impairs the physiological function of the adult retina.

## 3. Discussion

In the present study, first, our results clarified the question of whether Gata3 is expressed in the postnatal retina. Although Gata3 was expressed throughout the brain before P25 and weakly expressed in specific regions [4] that are part of the central nervous system, most studies suggested that Gata3 was not expressed in the retina from embryogenesis to adulthood. Gata3 expression was only strictly confined to the differentiating lens fiber cells of the embryonic eye [9,10]. However, our data revealed that Gata3 is expressed not only in a retinal precursor cell line but also in the postnatal retina, based on Q-PCR, immunohistochemical staining, and Western blot assays.

Second, regarding its bioactivity, Gata3 participates in maintaining the retinal precursor state, but not regenerative neurogenesis. Gata3 silencing is involved in the differentiation of retinal precursor cells. As shown in Figure 1 and Figure 2, silencing Gata3 with an siRNA significantly downregulated Nestin, a marker of stem cells, increased the expression of Map2, and promoted neurite outgrowth in 661W retinal precursor cells. Not only that, the increased expression of class III beta-tubulin (Tuj1, a neuron marker) and decreasing expression of pax6 (a neuronal progenitor marker) were also observed in Gata3-silenced 661W cells, further suggesting its role in neuron differentiation. (Figure 3). However, exogenous Gata3 did not induce cell proliferation in vivo (Figure 6C). These results are not consistent with a previous study. Kizil reported that re-expression of Gata3 induces regenerative neurogenesis in the zebrafish brain. A few newborn neurons were observed in the ventricular region of the telencephalon [12]. We speculated that this discrepancy is induced by the cell type. For example, our data showed that Gata3 silencing reduced the viability of proliferating 661W cells (Figure 1), whereas exogenous Gata3 did not increase the viability of nonproliferating primary retinal neurocytes (P1) (Figure 5G). Moreover, different levels of exogenous Gata3 might also have different outcomes. Moreover, more progenitor cells were observed in the ventricular zone of the adult zebrafish telencephalon [32,33]. Accordingly, these progenitor cells might exhibit increased proliferation upon Gata3 re-expression in the brain. Conversely, primary postnatal retinal neurons do not proliferate, indicating that exogenous Gata3 reduces cell viability and increases DNA damage (Figure 5). Similarly, fewer progenitor cells are present in the adult retina [34,35]. In the retina of normal adult mice, Nestin is rarely expressed except for some vascular profiles in the outer plexiform layer (OPL) [36,37]. Therefore, re-expression of Gata3 does not induce neurogenesis but activates apoptosis in the adult retina (Figure 7). Yet, the underlying mechanism of the exact role of Gata3 in DNA damages needs further investigation.

Third, a previous study suggested that Gata3 might be a potential therapeutic target for neural protection in the brain [38,39]. However, our data indicate that exogenous Gata3 significantly decreased synaptophysin levels and cell viability in vitro (Figure 5) and induced serious damage in the outer segment of photoreceptors (Figure 6). Exogenous Gata3 remarkably induced apoptosis in the ONL of retinas expressing Gata3 compared to the control. Accordingly, a- and b-waves were decreased, indicating that visual function was damaged in adult mice with exogenous Gata3 expression (Figure 7). Taken tother, Gata3 is associated with damage as a negative factor, which might not promote ocular neural recovery in individuals with visual neural diseases.

## 4. Materials and Methods

**Primary mouse retinal neurons culture and treatment.** Primary mouse retinal neurons were cultured as described previously [40]. Mice were provided by the animal center of Zhongshan Ophthalmic Center, Sun Yat-sen University, China. Briefly, 10 postnatal 1day mice were sacrificed. Retinas were separated from their eyeballs followed by incubation in 0.125% trypsin for 15 min at 37 °C to dissociate the tissues. After adding culture medium to terminate digestion, tissues were dissociated into single cells mechanically through a narrow-bore Pasteur pipette. The dissociated cells were seeded at a density of ~ 1 × 10^6^ cells per ml in poly-L-Lysine-coated culture plates. After 12 h the cells underwent 10 μmol/mL Ara-C (Sigma, Saint-Louis, MO, USA) treatment to prevent the proliferation of non-neurons. Then the cells were maintained in complete medium (DMEM supplemented with 10% FBS) and characterized by Map-2 staining (Boster, Wuhan, China BM1243, 1:100). Three days after culture, the cells were infected with Gata3 overexpression virus (AAV-Re-Gata3-T2A-GFP; Cyagen Biosciences, Santa Clara, USA) or control virus (AAV-Re-GFP; Cyagen Biosciences, Santa Clara, USA). Cells were cultured for another 3 days before immunostaining. All experiments were repeated at least three times.

**661W cell culture.** The mouse retinal cell lines 661W, purchased from ATCC (Manassas, VA, USA), were cultured in Dulbecco’s Modified Eagle’s Medium (DMEM, Gibco, CA, USA) supplemented with 10% fetal bovine serum (FBS; Gibco, CA, USA) and 1% penicillin/streptomycin (Gibco, CA, USA) in a humidified 5% CO2 incubator. Trichostatin A (TSA, Sigma, Saint-Louis, MO, USA) was added to a final concentration of 500 nM to induce cell differentiation. The control group was treated with an appropriate vehicle. After 48 h incubation, live cell images were obtained by a Zeiss Axio Observer Z1 microscope (Carl Zeiss Microscopy, Munich, Germany) with Axiovision Rel. 4.8 software (Carl Zeiss Imaging Solutions GmbH, Munich, Germany). Neurite outgrowth was then quantified using Photoshop CS6 v13 software (Adobe, San José, CA, USA), and more than 200 cells were analyzed for each group.

**Virus preparation**. Recombinant adeno-associated virus AAV-Re-GFP and AAV-Re-Gata3-T2A-GFP were produced and packaged into recombinant adeno-associated virus 2. Retro 26 by Cyagen Biosciences, using the mouse GATA binding protein 3 (GATA3) cDNA sequence (Accession number NM_001002295.2). A T2A element with self-cleaving ability was applied to achieve the co-expression of Gata3 and GFP in the AAV-Re-Gata3-T2A-GFP. AAV was stored in 150 mM NaCl, 2 mM MgCl2, 50 mM Tris (pH 8.0) at −80 °C for less than 1 year and thawed on ice on the day of use.

**Western Blotting.** Total protein of cells or tissues was extracted using radio-immuno-precipitation assay buffer supplemented with PMSF, followed by centrifuging the tubes at 4 ℃ for 15 min at 13,300 rpm to remove debris. The following primary antibodies were used: rabbit anti-Gata3 (Abcam, Cambridge, MA, USA, ab199428, 1:500), mouse anti-Map2 (Boster, Wuhan, China, BM1243, 1:1000), mouse anti-Nestin (Millipore, MA, USA, mab353, 1:1000), mouse anti-Synaptophysin (Abcam, Cambridge, UK, ab8049, 1:500), mouse anti-pax6 (DSHB, USA, AB528427, 1:500), mouse anti-Tuj1 (Abcam, Cambridge, UK, ab7751, 1:500). The membrane was incubated with horseradish peroxidase-conjugated secondary anti-rabbit (CST, Danvers, MA, USA, 7074s, 1:10,000) or anti-mouse antibody (CST, Danvers, MA, USA, 7076s, 1:10,000). Gapdh (Protein Tech Group, Wuhan, China, 10494-1-AP, 1:2000) served as a loading control. Protein bands were detected using an enhanced chemiluminescence detection system (Millipore, MA, USA). The WB samples came from single samples (*n* = 6).

**Immunofluorescence analysis**. Immunofluorescence assay was performed according to the standard protocol. In brief, cells were fixed with 4% paraformaldehyde after the treatment mentioned above. Following permeabilization by 0.1% Triton X-100, cells were blocked for 30 min with 10% goat serum. The primary antibodies were used as follows: mouse anti-Map2 (Boster, Wuhan, China, BM1243, 1:100), rabbit anti-Map2 (Abcam, Cambridge, UK, ab254264, 1:200), rabbit anti-Nestin (Millipore, MA, USA, mab353, 1:100), mouse anti-synaptophysin (Abcam, Cambridge, MA, USA, ab8049, 1:100), rabbit anti-Gata3 (Abcam, Cambridge, UK, ab199428, 1:100), mouse anti-pax6 (DSHB, Lowa, USA, AB528427, 1:100), mouse anti-Tuj1 (Abcam, Cambridge, UK, ab7751, 1:100). Cells were then incubated with secondary anti-mouse (CST, Danvers, MA, USA,4408s, 4409s, 1:500) or anti-rabbit antibodies (CST, Danvers, MA, USA, 4413s, 4412s, 1:500) for 1 h at room temperature. Dapi was used as a counterstain for nuclei and images were captured by obtained by a Zeiss Axio Observer Z1 microscope (Carl Zeiss MicroImaging GmbH, Munich, Germany) with Axiovision Rel. 4.8 software (Carl Zeiss Imaging Solutions GmbH, Munich, Germany).

**Immunohistochemical assay.** An immunohistochemical assay was performed on retinal slides of postnatal day1 and adult mice, according to the manufacturer’s protocols of SABC-POD (F) rabbit IgG kit (Boster, Wuhan, China). Rabbit anti-Gata3 (Abcam, Cambridge, UK, ab199428, 1:100), rabbit anti-Nestin (Millipore, MA, USA, mab353, 1:100) were used as primary antibodies, and biotinylated anti-rabbit IgG antibody was used as a secondary antibody. Following washing, the sections were developed with 3,3′-diaminobenzidine tetrahydrochloride (DAB) peroxidase substrate (Boster, Wuhan, China) and counterstained with hematoxylin. The images were obtained by a Zeiss Axio Observer Z1 microscope (Carl Zeiss MicroImaging GmbH, Munich, Germany) with Axiovision Rel. 4.8 software (Carl Zeiss Imaging Solutions GmbH, Munich, Germany).

**Cell viability assayed by CCK-8.** Cell viability was determined by a cell counting Kit-8 (CCK8) assay (Dojindo, Rockville, USA). Cells were incubated with CCK8 reagent for 2 h at 37 °C followed by measuring the optical density at 450 nm. Cell viability was normalized to the untreated control.

**RNA interference.** The siRNA targeting against Gata3 is a pool of three different sequences: Gata3-siRNA-1: 5′-GACGGAAGAGGUGGACGUA(dTdT)-3′; Gata3-siRNA-2: 5′-UCGUACAUGGAAGCUCAGU(dTdT)-3′; Gata3-siRNA-3: 5′-GAUUUCAGAUCUGGGCAAU(dTdT)-3′. The control siRNA is as follows: 5′-CCUACGCCACCAAUUUCGU(dTdT)-3′. The oligos were synthesized by Ribobio (Guangzhou, China). Transfections were performed with Lipofectamine RNAiMAX (Invitrogen, Waltham, MA, USA ) and the expression level of Gata3 was measured by Western blot at 24 h post-transfection.

**5-ethynyl-2′-deoxyuridine(EDU) and Propidium iodide (PI) Nucleic Acid Staining.** After RNA interference for 24 h, the 661W cells were treated with EDU (10 μM, A10044, Invitrogen) diluted by the media overnight. Then, the cells were treated with Propidium iodide solid (500 nM, P1304MP, Invitrogen) diluted by the media for 15min and immediately fixed with 4% paraformaldehyde. After washing 3 times with 2 × SSC (0.3 M NaCl, 0.03 M sodium citrate, pH 7.0), the Click-iT Edu Imaging Kit (C10337, Invitrogen, Waltham, MA, USA) was used to detect the EDU staining. Dapi was used to stain the nuclei and images were obtained by a Zeiss Axio Observer Z1 microscope (Carl Zeiss MicroImaging GmbH, Munich, Germany) with Axiovision Rel. 4.8 software (Carl Zeiss Imaging Solutions GmbH, Munich, Germany).

**Experiments in vivo.**

**Animals.** Wide-type adult C57BL/6J mice (6- to 8-weeks old) were obtained from Ophthalmic Animal Laboratory, Zhongshan Ophthalmic Center, Sun Yat-sen University. All the experiments were approved by the Institutional Animal Care and Use Committee of Zhongshan Ophthalmic Center (Permit Number: SYXK (YUE) 2010-0058). Mice were anesthetized with tribromoethanol (0.14 mL/10 g body weight of 1.25%). Phenylephrine HCl (0.5%) and tropicamide (0.5%) were used to dilate pupils. Using a glass micropipette, intravitreal injections were performed as previously described with 2 μL of AAV-Re-GFP or AAV-Re-Gata3-T2A-GFP (both were diluted to a titer of 1 × 10^12^ VG/mL with sterile PBS), respectively. Briefly, once animals were anesthetized and pupils were dilated, a small incision on the limbus area was made using a 30 G needle. A glass micropipette with a 30 G blunt needle performed the intravitreal injections through the limbal incision, avoiding touching the lens and directing the tip of the needle to the vitreous cavity around the optic nerve head. After injections, animals were treated topically on the cornea with neomycin and polymyxin b sulfates and put on a heated platform (37 ℃) to recover from anesthesia.

**Retinal sections and immunofluorescence**. Six eyes for each treatment group were immediately fixed in 4% paraformaldehyde overnight upon enucleation as eyecups, washed with PBS, and evaporated with gradient sucrose before being frozen in OCT (Sakura Finetechnical Co. Tokyo, Japan). Frozen sections (10 μm) were incubated with rabbit anti-Gata3 antibody (Abcam, Cambridge, UK, ab199428, 1:100) overnight at 4℃. A secondary anti-rabbit antibody (CST, MA, USA, 4413s, 1:500) was added at room temperature, and DAPI was used to stain the nuclei. Photomicrographs were captured by Zeiss Axio Observer Z1 microscope (Carl Zeiss MicroImaging GmbH, Munich, Germany) with Axiovision Rel. 4.8 software (Carl Zeiss Imaging Solutions GmbH, Munich, Germany).

**EDU staining.** Mice (*n* = 6 for each treatment group) were stained with EDU (A10044, Invitrogen, Waltham, MA, USA) by intraperitoneal injection (200 mg/kg), 12 h after the injection, the mice eyes were harvested and fixed in 4% paraformaldehyde overnight upon enucleation, then evaporated with gradient sucrose before being frozen in OCT (Sakura Finetechnical Co., Tokyo, Japan). Frozen sections (10 μm) EDU staining were detected by the Click-iT Edu Imaging Kit (C10340, Invitrogen). DAPI was used as a counterstain of nuclei and images were obtained by a Zeiss Axio Observer Z1 microscope (Carl Zeiss MicroImaging GmbH, Munich, Germany) with Axiovision Rel. 4.8 software (Carl Zeiss Imaging Solutions GmbH, Munich, Germany).

**Electroretinogram (ERG).** Mice (*n* = 6 for each treatment group) were dark-adapted for 12 h, in vivo retinal function was determined with the RETIscan system (Roland Consult, Wiesband, Germany) as described by Yang [41]. Briefly, under dim red-light, mice were anesthetized with tribromoethanol (0.14 mL/10 g bodyweight of 1.25%) and placed on a heated platform (37 ℃). Phenylephrine HCl (0.5%) and tropicamide (0.5%) were used to dilate pupils. Animals were stimulated with a green flash with an intensity of 3.0 cds/m^2^ under dark adaptation and recorded for the photopic response. ERG data were collected by the amplifier of the RETI-scan system at a sampling rate of 2 kHz, and subsequently analyzed with RETIport software (Roland Consult).

**Statistical analysis.** All in vitro experiments were performed at least 3 times. Data are expressed as the means ± SEM. The differences between mean values were evaluated with the two-tailed Student’s *t*-test (for two groups) and the analysis of variance (for 2 groups) and the analysis of variance (ANOVA, for > 2 groups). All calculations and statistical tests were performed by the computer programs Microsoft Excel 2003 (Microsoft, Redmond, WA, USA) or SPSS 11.5 (SPSS, Chicago, IL, USA). *p* < 0.05 was considered significant for all analyses.

## 5. Conclusions

The present study provides insights into the expression profile and bioactivity of Gata3 in the retina. Gata3 is likely to participate in maintaining the neural progenitor state. Gata3 silencing is involved in neuronal differentiation and cell viability. In addition, re-expression of Gata3 impedes the physiological function of adult retinal neurons.

## Data Availability

The datasets generated during and/or analyzed during the current study are not publicly available due to following study request, but are available from the corresponding author on reasonable request.

## Supplementary Materials

The following supporting information can be downloaded at: https://www.mdpi.com/article/10.3390/ijms23052495/s1.
