# Supplementary material for: Gata3 Silencing Is Involved in Neuronal Differentiation and Its Abnormal Expression Impedes Neural Activity in Adult Retinal Neurocytes
Source: Int J Mol Sci. 2022 Feb 24;23(5):2495. doi: 10.3390/ijms23052495 (PMC8910128; doi:10.3390/ijms23052495)
Supplement: Supplementary file 1 [file ijms-23-02495-s001.zip › ijms-1579923 supplementary.pdf]

Supplemental data 1

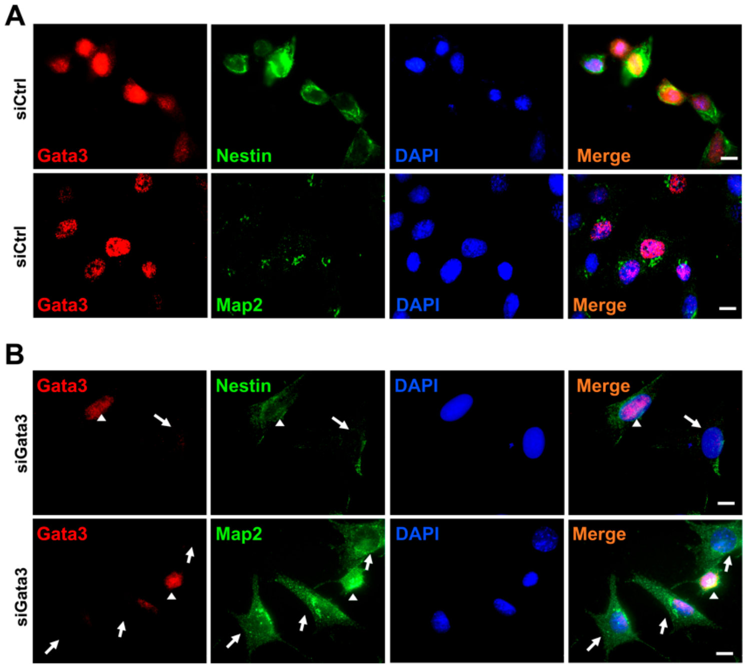

Supplemental data 1**.** Supplemental data 1. Double staining in 661W cells treated with siRNA. (**A**) Strong Nestin (green) and faint Map2 staining were observed in 661W cells treated with control siRNA. (**B**) Significantly decreased Nestin and increased Map2 staining (green) were observed in the Gata3-silenced cells, with distinguish neurites (white arrows). In the cells that still expressed Gata3 (red), poorly differentiated cells with both Map2 and Nestin positivity were observed (white arrowheads). The nucleus is labeled with DAPI (blue). Scale bars represent 10 µm.

Supplemental data 2

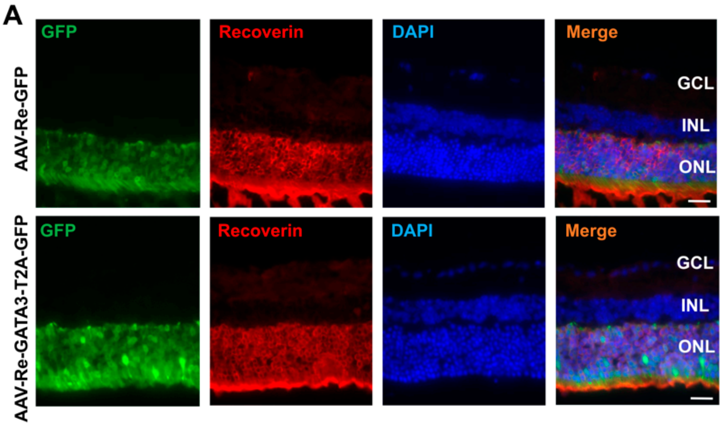

Supplemental data **2.** Supplemental data 2. Most GFP-positive cells in mouse retina were photoreceptors. Retina cells infected with adenovirus AAV-Re-GFP or AAV-Re-GATA3-T2A-GFP were labeled with GFP protein (green). Recoverin-labeled photoreceptor in the ONL of mouse retina (red). The overlaps staining presents orange. The nucleus is labeled with DAPI (blue). Scale bars represent 50 µm.
