# Supplementary figures and images for: Gata3 Silencing Is Involved in Neuronal Differentiation and Its Abnormal Expression Impedes Neural Activity in Adult Retinal Neurocytes
Source: Int J Mol Sci. 2022 Feb 24;23(5):2495. doi: 10.3390/ijms23052495 (PMC8910128; doi:10.3390/ijms23052495)

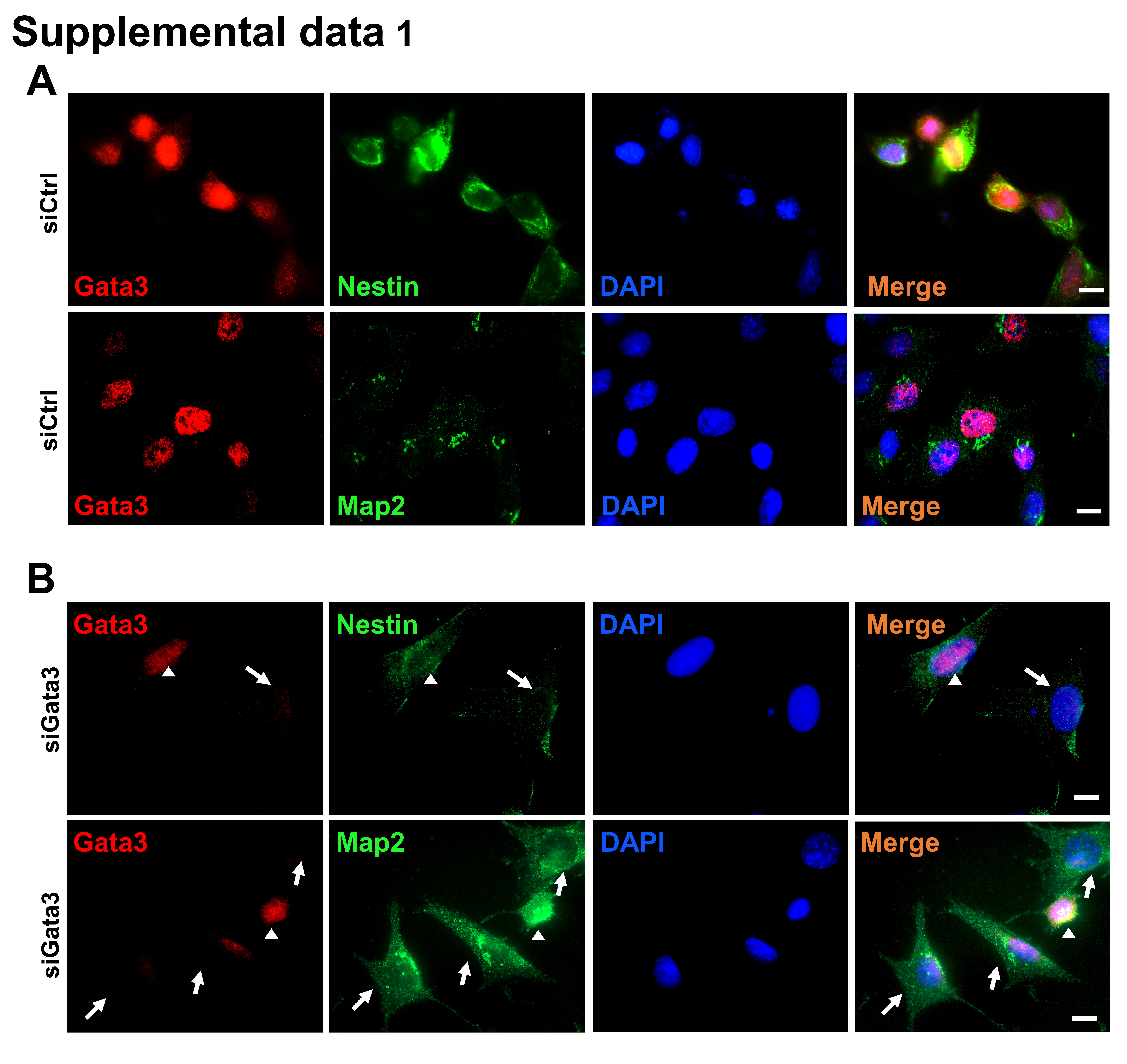

Supplement: Supplementary file 1 [file ijms-23-02495-s001.zip › Supplemental data 1.tif]

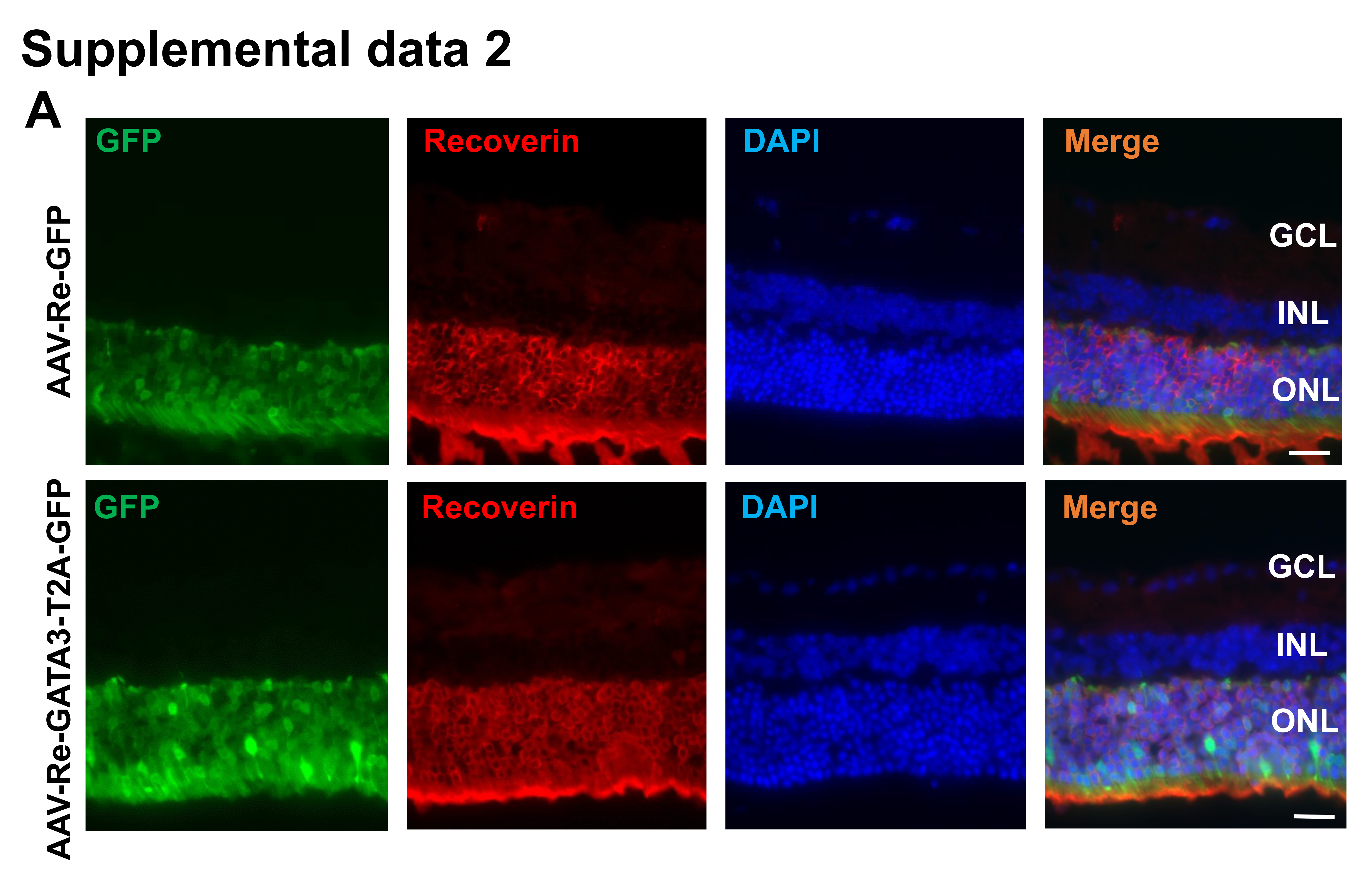

Supplement: Supplementary file 1 [file ijms-23-02495-s001.zip › Supplemental data 2.tif]
